# Treatment regimens for optimising outcomes in patients with neovascular age-related macular degeneration

**DOI:** 10.1038/s41433-024-03370-0

**Published:** 2024-10-08

**Authors:** Kelvin Yi Chong Teo, Bora Eldem, Antonia Joussen, Adrian Koh, Jean-François Korobelnik, Xiaoxin Li, Anat Loewenstein, Monica Lövestam-Adrian, Rafael Navarro, Annabelle A. Okada, Ian Pearce, Francisco Rodríguez, David Wong, Lihteh Wu, Dinah Zur, Javier Zarranz-Ventura, Paul Mitchell, Varun Chaudhary, Paolo Lanzetta

**Affiliations:** 1 Singapore National Eye Centre, Singapore, Singapore; 2 Department of Ophthalmology, Hacettepe University, School of Medicine, Ankara, Turkey; 3 Charité – University Medicine Berlin, Berlin, Germany; 4 Camden Medical Centre, Singapore, Singapore; 5 Service d’ophtalmologie, CHU Bordeaux, Bordeaux, France; 6 University of Bordeaux, INSERM, BPH, UMR1219, F-33000, Bordeaux, France; 7 Xiamen Eye Center, Xiamen University, Xiamen, China; 8 Division of Ophthalmology, Tel Aviv Sourasky Medical Center, Sackler Faculty of Medicine, Tel Aviv University, Tel Aviv, Israel; 9 Department of Ophthalmology, Lund University Hospital, Lund, Sweden; 10 Retina and Vitreous Department, Institute of Ocular Microsurgery, Barcelona, Spain; 11 Department of Ophthalmology, Kyorin University School of Medicine, Tokyo, Japan; 12 Royal Liverpool University Hospital, Liverpool, UK; 13 Fundación Oftalmológica Nacional, Escuela de Medicina y Ciencias de la Salud, Universidad del Rosario, Bogotá, Colombia; 14 Unity Health Toronto – St. Michael’s Hospital, University of Toronto, Toronto, ON Canada; 15 Macula, Vitreous and Retina Associates of Costa Rica, San José, Costa Rica; 16 Hospital Clínic de Barcelona, University of Barcelona, Barcelona, Spain; 17 Department of Ophthalmology, Centre for Vision Research, Westmead Institute for Medical Research, the University of Sydney, Sydney, Australia; 18 Hamilton Regional Eye Institute, St. Joseph’s Healthcare Hamilton, Hamilton, ON Canada; 19 Department of Surgery, McMaster University, Hamilton, ON Canada; 20 Department of Medicine – Ophthalmology, University of Udine, Udine, Italy

**Keywords:** Macular degeneration, Retinal diseases

## Abstract

Practice patterns for neovascular age-related macular degeneration (nAMD) have evolved from the landmark registration trials of vascular endothelial growth factor (VEGF) inhibitors. Non-monthly regimens like treat-and-extend (T&E) have become popular due to their effectiveness in clinical practice. T&E regimens attempt to limit the burden of visits and treatments by allowing progressively longer treatment intervals, but in so doing, are potentially associated with the expense of treating quiescent disease. This is acceptable to many patients and their ophthalmologists but can still be problematic in the real-world. Recent studies have further refined the T&E approach by allowing for quicker and longer extension of treatment intervals when less severe disease is detected. With newer drugs offering increased durability, a shift to longer regular intervals may emerge as a new practice pattern for VEGF inhibitor therapy. This review aims to consolidate the current literature on the most effective treatment patterns and update treatment guidelines based on options that are now available. It also summarises new aspects of nAMD management that may help to further refine current practice.

## Abstract (zh)

新生血管性年龄相关性黄斑变性(nAMD)的治疗模式已从具有里程碑意义的血管内皮生长因子 (VEGF) 抑制剂注册试验中进行转变。治疗并延长方案(T&E)等非每月治疗方案因其在临床实践中的有效性而被广泛应用。T&E方案旨在通过逐渐延长治疗间隔来减轻就诊和治疗的负担, 但可能增加静止期疾病的治疗费用。这对许多患者及眼科医生来说可以接受, 但在真实世界中实施仍存在困难。最近的研究进一步完善了T&E方案, 当检测到病情较轻时, 可以更快、更长地延长治疗间隔。随着新药物提供了更高的持久性, 转向更长的规律间隔将成为VEGF抑制剂治疗的新的临床模式。本文旨在综合目前关于有效治疗模式的文献, 并根据现有的有效方案更新治疗指南。本文还总结了nAMD管理的新的进展, 有助于进一步完善当前的临床实践。

## Introduction

Treatment for neovascular age-related macular degeneration (nAMD) has evolved rapidly over the past decade, during which pivotal trials have established the efficacy of vascular endothelial growth factor (VEGF) inhibitor therapy in this indication [1–3]. However, outcomes in clinical settings are inferior to those reported in clinical trials [4, 5], likely as a result of undertreatment, poor adherence and inappropriate treatment decisions [6–8].

Guidance on treatment has previously been proposed but, with ongoing changes in the treatment landscape, it is timely to consider a set of current, pragmatic, clinically applicable guidelines that can close the gap between clinical trials and real-world outcomes. This review aims to summarise the key evidence from both clinical trials and real-world studies to update previous recommendations and provide a treatment framework that is useable in current clinical practice.

## Methods

This article is based on a review of the literature and a consensus among retinal experts from the Vision Academy. The Vision Academy is a group of over 100 international experts who, through their collective expertise, provide consensus guidance for managing clinically challenging situations, especially in areas of controversy or with insufficient conclusive evidence (www.visionacademy.org). The Vision Academy is sponsored by Bayer.

The current article provides an update to the ‘Fundamental principles of an anti-VEGF treatment regimen’ paper published in 2017 [9]. Having identified the need for updated recommendations on the use of VEGF inhibitor therapy for nAMD, the online PubMed database was searched for relevant articles published in the English language between 2017 and 2021. Relevant articles and data published during manuscript development were also incorporated. The recommendations presented in this paper, which take into account the latest evidence relating to pharmaceutical products and treatment regimens, were developed by the authors and subsequently reviewed, commented on, and endorsed by a majority of the Vision Academy. For each proposed recommendation, respondents were asked to rate their agreement using a 5-point categorical scale: ‘strongly agree’, ‘agree’, ‘neither agree nor disagree’, ‘disagree’ and ‘strongly disagree’. Responses from more than 50% of the Academy were required for the survey to be valid. To assess any influence of the healthcare system on the survey responses, respondents were additionally asked for the reimbursement status of treatment in their country of practice (reimbursed, ‘out-of-pocket’ or a combination of the two). Biases were assessed using χ^2^. Endorsement was established if 50% or more of respondents indicated that they agreed or strongly agreed with a recommendation; consensus was considered “strong” if greater than 75% of respondents agreed or strongly agreed. The list of Vision Academy members and mentees who contributed to the recommendations is provided at the end of this article.

## Evidence and recommendations for the treatment of nAMD with VEGF inhibitors

Recommendations for the treatment of nAMD, with supporting evidence, are detailed below. The recommendations are divided according to the phase of treatment, allowing the physician to follow the recommendations and the supporting evidence chronologically over the course of a patient’s treatment journey. The recommendations were formulated by the authors of the manuscript and submitted to the entire Vision Academy membership for endorsement; 55 responses (including from the authors) were received. Overall, the recommendations were endorsed by 95.5% of the respondents (a response of ‘agree’ or ‘strongly agree’), with the level of endorsement for each individual recommendation ranging from 89.1% to 98.2%. The mean (range) rate of non-endorsement was 3.2% (1.8–5.5%) for a response of either ‘disagree’ or ‘strongly disagree’, and 1.4% (0–5.5%) for a response of ‘neither agree nor disagree’. (It should be noted that these recommendations represent the ideal scenario, and full implementation may not be possible in all clinics).

### Early treatment phase

#### Early intervention

On establishing the diagnosis of nAMD, early commencement of VEGF inhibitor therapy has been found to maximise visual outcomes. A prolonged delay between the first reported symptoms and the first administration of VEGF inhibitor therapy has been found to be a significant predictor of poor visual outcomes, with a 2.6-fold increase in the risk of poor vision with a delay of >21 weeks compared to >7 weeks [10]. Studies of nAMD in fellow eyes show considerably better outcomes when treatment is commenced early because the fellow eyes benefit from opportunistic monitoring during visits intended for the affected eye [11–13]. Early, favourable response is also associated with improved visual outcomes for up to 3 years [14]. Early intervention is, however, contingent on patients presenting with early disease, which may go unnoticed if their vision remains good [15]. Strategies to mitigate the risk of early disease being missed include the monitoring of high-risk eyes or the use of novel models of care, such as self-monitoring [16–20]. Innovative deep-learning techniques applied to retinal multimodal imaging may also help to predict the time to first onset of treatable disease [21–25].

#### Early intensive treatment

Early intensive treatment has also been associated with favourable outcomes [26]. A retrospective analysis from the UK found greater visual improvements in patients who underwent a loading phase at the start of treatment (the first three doses received within 90 days) versus those who did not [27]. Eyes with treatment intervals of more than 5 weeks initially required a much longer time to achieve disease quiescence compared with those that received 4-weekly treatment, and required twice as many injections over time [28].

##### Recommendation 1: Early intensive treatment to maximise visual outcomes

Treatment should commence as soon as disease activity is detected; however, this can be challenging as patients may not be aware that their symptoms herald a more serious condition. Early intensive treatment should be considered to achieve disease quiescence rapidly and maximise visual outcomes in the long term.

### Treatment regimen after the loading phase

The high treatment burden of monthly treatments as per landmark trials is untenable in real-world practice. Subsequent trials were designed specifically to overcome this clinical challenge by evaluating the efficacy of non-monthly or variable treatment regimens.

#### Quarterly dosing

In the PIER (Phase 3b, multicentre, randomised, double-masked, sham injection-controlled study of the efficacy and safety of ranibizumab in subjects with subfoveal CNV with or without classic CNV secondary to AMD) study where treatment was administered quarterly, vision in the ranibizumab 0.3 mg and 0.5 mg groups decreased by 1.6 and 0.2 ETDRS letters, respectively, from baseline to 12 months [29]. The EXCITE (Efficacy and Safety of Ranibizumab in subjects with Subfoveal and CNV secondary to AMD) study, which compared quarterly versus monthly dosing, reported vision gains of 4.0 versus 8.0 ETDRS letters [30].

Non-fixed-dose regimens, which are designed to personalise nAMD treatment, include two treatment strategies in use today: the as-needed, or *pro re nata* (PRN), and the treat-and-extend (T&E) regimens [31–35]. A key component of non-monthly regimens is the assessment of disease activity. Most studies use a combination of visual acuity and anatomical features to assess the status of disease; these can vary from study to study, but generally follow similar principles. Signs of disease activity include vision loss related to the disease, the presence of intraretinal fluid and/or subretinal fluid (SRF) detected on optical coherence tomography (OCT), and increased or new haemorrhage [2, 33, 36–39].

#### As-needed (*pro re nata*) regimens

The PRN treatment strategy is a reactive approach to therapy and dictates that treatment be administered when the disease is active and withheld when it is quiescent. Assessment must be performed monthly, which contributes to the overall burden of clinical care and may be untenable in practice [40]. While outcomes were equivalent with PRN and fixed dosing in the CATT study [41], a meta-analysis showed that outcomes with the PRN approach were consistently inferior to those with either fixed or T&E regimens in both clinical trials and real-world settings [5].

#### Treat-and-extend regimens

The T&E strategy is a proactive approach that requires treatment to be administered at every visit even if the disease is quiescent, with the subsequent treatment interval being determined by the disease activity status. T&E allows for longer treatment intervals during periods of disease quiescence but requires the patient to receive treatment despite quiescent disease. The T&E regimen investigated in early trials used a 2-week treatment interval extension up to a maximum of 12 weeks if the disease was continuously quiescent. The treatment interval was reduced by 2 weeks from the previous interval if disease activity was detected [36–38].

The three landmark trials that established the T&E regimen as a viable treatment strategy were the TREX [42], TREND [37] and CANTREAT [36] studies. These studies compared T&E versus monthly regimens and found no significant difference in visual outcomes. The TREX, TREND and CANTREAT studies reported non-significant differences of 1.8 [42], −1.9 [37] and 0.9 [36] letters respectively between groups, and a subsequent meta-analysis including TREX and TREND reported similar findings [43]. The number of treatments was significantly reduced in the T&E versus the monthly arms in all studies (Tables 1 and 2) [36–38].

**Table 1:** Summary of key trials that compared vision outcomes with three different regimens: monthly, PRN^a^, and T&E^b^.

	HARBOR [85]	CATT [86]	IVAN [39]	TREND [37]	TREX [42]	ALTAIR [52]
Duration, years	1	2	1	1	2	2
Mean VA change, ETDRS letters, monthly regimen^c^	10.1	8.8 ± 15.9	6.1 ± 14.0	8.1 ± 10.6	10.5 ± 14.1	-
Mean VA change, ETDRS letters, PRN regimen^c^	8.6	6.7 ± 4.6	5.0 ± 11.1	-	-	-
Mean VA change, ETDRS letters, T&E regimen (2-weekly extensions)^c^	-	-	-	6.2 ± 4.5	8.7 ± 7.3	7.6 (5, 10.3)^d,e^
Mean VA change, ETDRS letters, T&E regimen (4-weekly extensions)	-	-	-	-	-	6.1 (3.1, 9.0)^d,e^
Number of treatments in the T&E/PRN arm, mean (SD)	6.9 (2.4) ranibizumab 2 mg PRN	12.6 (6.6) ranibizumab 0.5 mg PRN	10 (6, 12)^f^ ranibizumab 0.5 mg PRN	8.7 (2.7) ranibizumab 0.5 mg T&E	10.1 (7–13)^g^ ranibizumab 0.5 mg T&E	10.4 (2.6) with 2-week extension, aflibercept 2 mg 10.4 (2.4) with 4-week extension, aflibercept 2 mg

^a^Monthly visits to monitor lesion activity, and treatment administered only when lesion is active.

^b^Treatment is administered at every visit but the interval between visits varies depending on lesion activity.

^c^Expressed as mean letter score ± SD. In the HARBOR study only, expressed as mean letter score.

^d^Expressed as mean letter score (95% CI).

^e^Retreatment regimen in the ALTAIR study also allowed for maintenance of the treatment interval if the residual fluid had decreased from the previous visit, even with persistent fluid.

^f^Expressed as median (1st, 3rd quartile).

^g^Expressed as mean (range).

*ETDRS* Early Treatment Diabetic Retinopathy Study, *PRN* pro re nata (as-needed), *T&E* treat-and-extend, *VA* visual acuity.

**Table 2:** Summary of recent meta-analyses comparing T&E and alternative regimens.

Study	Comparisons	Duration, months	Design of included studies	Vision outcomes	Treatment load	Remarks
Okada, 2018 [43]	T&E vs PRN (2 studies)	12	RCT	Favours T&E +6.18 letters (95% CI: 3.28, 9.08)	T&E: more injections (1.44 more injections in T&E vs PRN)	T&E was found to be comparable to monthly (with fewer injections) and superior to PRN dosing for both vision gains and safety outcomes when using ranibizumab
	T&E vs monthly (2 studies)	24		Similar −1.79 letters (95% CI: 3.7, 0.13)	T&E: fewer injections (−1.6 [12 months] and −6.9 [24 months])	
Fallico, 2021 [87]	T&E vs monthly (4 studies)	12	RCT	Similar SMD = 0.08 (95% CI: −0.20, 0.35)	T&E: fewer injections SMD = −1.11 (95% CI: −1.67, −0.56)	T&E was comparable to a fixed monthly regimen throughout a 24-month follow-up and with a lower number of injections
	T&E vs monthly (2 studies)	24		Similar SMD = 0.04 (95% CI: −0.13, 0.21)	T&E: fewer injections SMD = −1.34 (95% CI: −1.54, −1.15)	
Li, 2020 [48]	T&E vs monthly (3 studies)	12	RCT	Similar +0.5 letters (95% CI: −3.1, 4.2)	T&E: fewer injections –2.4 injections (95% CI –2.7, –2.1)	The T&E group was sub-analysed as part of a larger PRN group. The authors concluded that while monthly regimens were more effective than PRN (whole group), the difference was small and clinically insignificant
Kim, 2016 [88]	T&E vs PRN (42 studies)	12	RWE	Favours T&E (+8.8 letters vs +3.5 letters with PRN)	T&E: more injections (6.9 injections vs 4.7 with PRN)	Although more injections were administered in the T&E group, fewer visits were needed. The authors concluded that over the long term, visual outcomes are dependent on the frequency of injections
		24		Favours T&E (+6.7 letters vs +1.3 letters with PRN)	-	
		36		Favours T&E (+5.4 letters vs −1.9 letters with PRN)	-	

*PRN* pro re nata (as-needed), *RCT* randomised controlled trial, *RWE* real-world evidence, *SMD* standardised mean difference, *T&E* treat-and-extend.

In terms of real-world data, an observational study conducted in Switzerland showed 8- and 5-letter gains at Year 1 and 2 respectively, with a mean of 8.3 aflibercept injections administered during Year 1 and 5.4 aflibercept injections administered during Year 2 [44]. The Fight Retinal Blindness! registry reported a gain of 8 letters over 24 months with a mean of 14 injections in patients on a T&E regimen [26], with most eyes maintaining vision gains for up to 3 years [45]. Longer-term results demonstrated an improvement of almost 2 lines at 6 years where patients were able to persist with T&E [46]. An observational study using the Fight Retinal Blindness! platform compared T&E versus fixed bimonthly regimens in patients with nAMD, and found similar clinical outcomes and median number of injections with the two approaches at 12 months; however, T&E resulted in a wider distribution of injection frequencies [47].

#### T&E versus PRN

A systematic review of 62 PRN and 8 T&E studies found better outcomes with T&E (10-letter gain with 8.1 injections) versus PRN regimens (5.4-letter gain with 5.6 injections) [48]. A recent meta-analysis also demonstrated better outcomes with T&E versus the PRN approach in clinical trials and real-world settings, as well as across different agent types [5].

#### Tailored T&E regimens

**Fig. 1:**
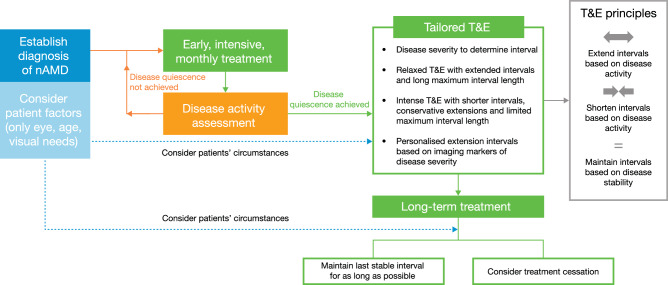
Algorithm for the treatment of nAMD. nAMD neovascular age-related macular degeneration, T&E treat-and-extend.

**Fig. 2:**
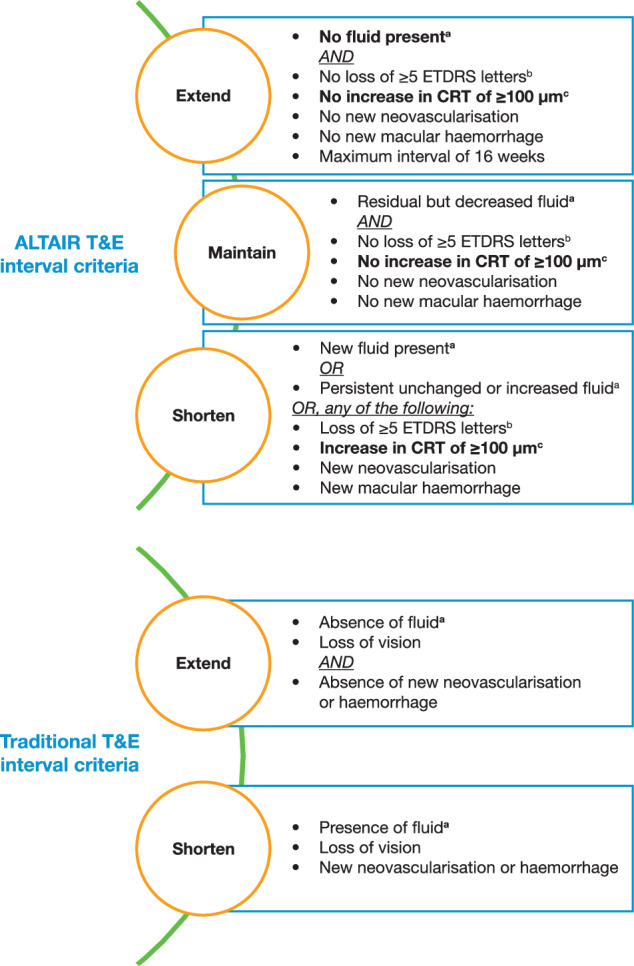
T&E interval criteria: ALTAIR versus traditional. ^a^As assessed by OCT. ^b^Loss of ≥5 ETDRS letters from the last treatment visit in conjunction with recurrent fluid on OCT. ^c^Increase in CRT of ≥100 μm compared with the lowest previous value on OCT. CRT central retinal thickness, ETDRS Early Treatment Diabetic Retinopathy Study, IRF intraretinal fluid, nAMD neovascular age-related macular degeneration, OCT optical coherence tomography, SRF subretinal fluid, T&E treat-and-extend.

Several clinical trials aimed to modify the “traditional” T&E regimen (2-week steps, with a 12-week maximum interval) by allowing for intervals to be extended or maintained at their current length, depending on disease severity. This “tailored” T&E regimen is included in our algorithm recommended for the treatment of nAMD (Fig. 1). A notable difference in these “tailored” versus “traditional” T&E regimens was the differentiation of SRF as a marker of reduced disease severity (Table 3). While any fluid (subretinal or intraretinal) was previously considered a sign of disease activity that required treatment intervals to be shortened, recent evidence has shown that stable SRF may be tolerated [49]. This was demonstrated in the FLUID study, which allowed intervals to be extended in the presence of SRF only [50], and the ARIES trial, which demonstrated good outcomes by allowing for interval extension if SRF did not exceed a thickness of 50 µm [51]. Another notable difference between “tailored” and “traditional” T&E regimens is the increased length of treatment intervals and the longest permissible interval. The ALTAIR trial studied a 4-week step allowing for intervals of up to 16 weeks [51, 52]. The study design also allowed for intervals to be (1) extended if no fluid was detected, (2) maintained if fluid was present but decreasing, and (3) shortened if there was persistent or unchanged fluid (Fig. 2). These varying treatment intervals and stratification of disease severity meant that patients could be treated with a much more personalised regimen than could have been achieved with the traditional T&E strategy.

**Table 3:** Definitions of disease activity in different trials.

Study	Treatment protocol	Criteria for lesion activity	Follow-up investigations
CATT [86]	PRN	• Fluid on OCT • New or persistent haemorrhage • Decreased VA compared with the previous examination • Dye leakage or increased lesion size on FA	• Monthly TD-OCT • FA at the discretion of the ophthalmologist
IVAN [39]	PRN	• Any SRF or increasing IRF on OCT • Fresh blood • VA loss of 10 letters • Fluorescein leakage 25% of the lesion circumference or expansion of CNV	• Monthly OCT • Monthly fundus photo • FA at the discretion of the ophthalmologist
GEFAL [2]	PRN	• Loss of 5 letters with no obvious atrophy or subretinal fibrosis and with fluid on OCT • Active exudation on OCT (SRF unless stable since the last 3 monthly injections, macular oedema with IRF, or increase in central subfield macular thickness of at least 50 μm compared with the previous examination) • Increased CNV area or persistence of leakage on FA • New or persistent subretinal or intraretinal macular haemorrhage	• Monthly TD/SD-OCT • Monthly fundus photo • FA and/or ICGA at the investigator’s discretion (mandatory only at baseline and 12 months)
HARBOR [85]	PRN	• Five-letter decrease in VA from the previous visit • Any evidence of disease activity on SD-OCT	• Monthly SD-OCT • FA and fundus photography at baseline and at Months 3, 6 and 12
ALTAIR [52]	T&E	• New or persistent fluid or increased fluid volume from visit as indicated by OCT • Loss of ≥5 letters from the previous visit with recurrent fluid on OCT • An increase in CRT of ≥100 μm at the central 1 mm compared with the lowest previous value on OCT • New-onset neovascularisation as determined at the investigator’s discretion • New macular haemorrhage	• SD-OCT
TREND [37]	T&E	• SD-OCT according to the investigator’s assessment (presence of IRF or SRF)	• SD-OCT
TREX [72]	T&E	• IRF and SRF on SD-OCT • Subretinal and intraretinal haemorrhage	• SD-OCT

*CNV* choroidal neovascularisation, *CRT* central retinal thickness, *FA* fluorescein angiography, *ICGA* indocyanine green angiography, *IRF* intraretinal fluid, *OCT* optical coherence tomography, *PRN* pro re nata (as-needed), *SD-OCT* spectral-domain optical coherence tomography, *SRF* subretinal fluid, *T&E* treat-and-extend, *TD-OCT* time-domain optical coherence tomography, *VA* visual acuity.

Novel automated algorithms have been developed to evaluate retinal fluid volumes to the level of nanolitres, which can be used as a new marker of disease severity [53–57] (in addition to SRF) to guide the ongoing treatment of nAMD [58].

The value of longer treatment intervals was particularly apparent during the COVID-19 pandemic, which led to severe restrictions on healthcare provision, with impacts on treatment for nAMD [59–61]. As a result, strategies have evolved to centre on control of the disease, including the introduction of “treatment only” visits and the extension of treatment intervals in cases of stable disease [62–64]. Table 2 summarises the current evidence from various meta-analyses on the use of the T&E regimen.

#### Observe and plan

The “observe and plan” strategy aims to reduce the number of monitoring visits by attempting to predict the regularity of treatment needs. This approach is intended to address the frequent monitoring visits borne by patients on PRN regimens, as well as the potential overtreatment of patients on T&E regimens. Briefly, the stable treatment intervals are determined by monthly evaluations, and treatment is administered at that interval without intervening monitoring visits for 2–3 cycles. A reassessment is performed following these cycles to enable interval adjustment for ongoing treatment [65].

#### VEGF inhibitor agents and regular extended intervals

The choice of agent is often dependent on physician preference, which, in turn, can depend on disease subtype and/or the financial status of the patient and prevailing healthcare reimbursement rules. For example, some physicians may choose to commence treatment with aflibercept in patients with polypoidal choroidal vasculopathy, especially if photodynamic therapy with verteporfin is not available. Objectively, this could be due to the existing evidence suggesting that aflibercept may be equally effective as monotherapy as when coupled with photodynamic rescue therapy [66].

An agent’s durability of action can be another consideration in treatment selection. This durability is a product of both the drug’s half-life and drug clearance by the individual patient. If the half-life can be accurately determined, physicians may be able to administer agents suited to extended treatment intervals specific to individual patients. A recent mathematical modelling study found that an unbound VEGF level below 0.001% is required to prevent clinical manifestations of disease and vision loss and that this level can be maintained with fixed 12-weekly dosing of aflibercept, compared with 8-weekly dosing of either ranibizumab or bevacizumab or 10-weekly dosing of brolucizumab [67].

In clinical trials, brolucizumab and faricimab have demonstrated the potential to support extended regular treatment intervals of 12 weeks and 16 weeks, respectively [3, 68]. At these intervals, the treatment burden is considerably reduced compared with monthly or bimonthly injections. The TENAYA and LUCERNE trials established non-inferiority between faricimab 6 mg administered at intervals of up to 16 weeks and aflibercept 2 mg administered every 8 weeks. Nearly 80% of patients in these two Phase 3 trials were able to complete treatment on intervals of at least 12 weeks, with around 45% completing on 16-week intervals [68]. It should be noted, however, that extended intervals of these lengths are not suitable for all patients, and that they require disease activity monitoring visits, which may not be easy to implement in clinical practice [3, 68]. Nonetheless, the above studies demonstrated the feasibility of longer regular intervals for the majority of patients [3, 68].

New formulations of established drugs, such as aflibercept 8 mg, may also provide greater efficacy and/or durability than currently available options. In the Phase 2 CANDELA trial, a greater proportion of eyes treated with aflibercept 8 mg versus 2 mg achieved a fluid-free centre subfield at Week 16 (51% versus 34%) [69]. In the follow-up Phase 3 PULSAR trial, mean increases of 5.6 and 5.5 letters were observed at Week 96 in patients treated with aflibercept 8 mg at 12- and 16-week intervals, respectively, with 88% of patients reaching a last assigned dosing interval of ≥12 weeks, 71% of patients reaching an interval of ≥16 weeks and 47% of patients reaching an interval of ≥20 weeks at the end of the 2-year study. The vision gains achieved with these extended intervals were similar to the 6.6-letter gain in patients treated with aflibercept 2 mg at 8-week intervals [70].

While new agents and formulations show potential for increased efficacy and durability and could change the landscape of treatment for nAMD, caution should be exercised in their use, and close monitoring for adverse events is necessary.

##### Recommendation 2: A treat-and-extend regimen should be commenced after lesion quiescence is achieved

Current evidence suggests that T&E is the most balanced treatment strategy in terms of good visual outcomes versus treatment burden [5, 71, 72]. T&E regimens allow for forward planning of visits but at the expense of potential overtreatment. Globally, many ophthalmologists have pivoted to T&E regimens to mitigate a high treatment burden [73].

In some cases, nAMD lesions can be aggressive and disease quiescence cannot be achieved despite regular and frequent treatments over an extended period [74–76]. These eyes fall broadly into the category of refractory/treatment-resistant nAMD.

Strategies that have been shown to be effective in these eyes include switching anti-VEGF agents or increasing VEGF dose [77], while evidence for other strategies, such as increasing dosing frequency, is limited [78].

##### Recommendation 3: Tailored treatment interval extensions

When commencing a T&E regimen after initial disease quiescence, treatment intervals can be tailored according to disease severity rather than the presence of disease activity. Disease severity can be contingent on fluid type and the nature of the disease, and its assessment may incorporate newer computational imaging techniques in the future. Despite good outcomes in patients treated with extended longer intervals and tolerance of some disease activity, more intensive treatment may be considered for patients treated for nAMD in their only seeing eye.

### Long-term treatment and treatment cessation

Real-world evidence is key to making informed decisions about long-term treatment and cessation, as it is impractical and costly to continue randomised controlled trials over several years. Long-term results from real-world studies have shown that most eyes are able to maintain vision gains on a T&E regimen after 2 years [45]. A recent report of 10-year real-world outcomes from two regions applying different treatment regimens found that patients in Australia and New Zealand, where a T&E strategy was followed, maintained their vision at baseline levels; by contrast, patients receiving PRN treatment in Switzerland lost approximately 15 letters [79]. This difference could have been due to eyes on the T&E regimen receiving more injections than those following the PRN strategy, resulting in better control of disease activity.

#### Recommendation 4: Long-term treatment and suspension

Treatment should be continued for as long as it remains tolerable to the patient. Long intervals between treatments can be considered in quiescent disease states, to allow background control of the disease. When vision is good, treatment suspension may be attempted in consultation with the patient, but close follow-up with OCT monitoring should be performed to ensure timely treatment if disease reactivation occurs. Treatment suspension should be strongly considered in patients where further treatment is futile, and where no further gains in vision are possible [80]. The status of the fellow eye is also important when considering treatment suspension. Caution should be exercised when considering suspension in cases where the better-seeing eye is undergoing treatment and end-stage AMD has developed in the other eye.

### Practical and novel aspects of initiating T&E in a clinical setting

T&E regimens consist of two main components: the assessment of disease severity, which results in a decision regarding treatment interval length, and administration of the treatment. These two components can be performed in the same visit (one-stop) or separate visits (two-stop). One-stop delivery is preferred; however, this can result in long visit times and constraints in terms of clinic resources. A two-stop model results in a shorter visit time but patients are required to attend more visits. Another consideration is bilateral treatment on the same day if both eyes are affected, which can significantly reduce the treatment burden [81]. There is, however, a risk that treatment intervals for the second eye are extended more quickly than would otherwise be the case, to match the intervals for the first affected eye, which are often longer than those for the second eye. This has been shown to be detrimental to long-term vision outcomes in the second affected eye and should therefore be avoided [82]. Eyes should be considered and treated individually, defaulting to the eye with the shorter interval in the case where patients are keen to reduce visits.

The use of virtual clinics for healthcare has become increasingly acceptable following the COVID-19 pandemic [83]. This model of care is also applicable to the management of nAMD, especially in cases where only monitoring is necessary [84]. In cases where treatments are required at every visit in a T&E regimen, the virtual clinic model of care can be considered where assessments and treatment can be performed on the same day, and the decision for the next interval can be provided asynchronously by the physician at a later time.

## Discussion and conclusions

In this article, we provide evidence and practical recommendations for the management of nAMD in today’s paradigm of VEGF inhibitor therapy. Some of the recommendations serve to affirm current practices, whilst others offer new insights that may change practice patterns. The departure from considering disease activity as binary, and accepting the concept of disease severity, can result in greater personalisation of treatment intervals. Disease severity can be measured as various aspects, including the quantification of fluid, fluid in different retina compartments, and even the location of fluid. This has become apparent with trials like PULSAR, which tolerated non-foveal fluid as part of the retreatment criteria. With greater understanding of the disease, we can determine aspects that may or may not affect functional outcomes. With new treatments on the horizon, the treatment landscape for nAMD will continue to evolve. The continued use of both clinical trials and real-world evidence will become even more important to ensure that the most effective treatments are chosen for clinical practice.
